# Investigating active ingredients in a complex intervention: a nested study within the Patient and Decision Aids (PANDAs) randomised controlled trial for people with type 2 diabetes

**DOI:** 10.1186/1756-0500-7-347

**Published:** 2014-06-07

**Authors:** Ian Brown, Alastair Bradley, Chirk Jenn Ng, Brigitte Colwell, Nigel Mathers

**Affiliations:** 1 School of Nursing, Midwifery and Social Work, University of Manchester, M13 9PL Manchester, UK; 2 Academic Unit of Primary Medical Care, University of Sheffield, Sheffield, UK; 3 Department of Primary Care Medicine, University of Malaya, Kuala Lumpur, Malaysia

**Keywords:** Patient decision aid, Process evaluation, Type 2 diabetes, Primary care

## Abstract

**Background:**

Randomised trials provide evidence that patient decision aids improve outcomes with respect to patient knowledge, involvement and satisfaction in decision making. It is less clear how these complex interventions are implemented within patient-clinician interactions and which components are active for improving decision processes. To investigate the experiences of using a diabetes treatment decision aid and to explore how components within a complex intervention influenced the decision making process.

**Methods:**

A pragmatic mixed methods study nested within the PANDAs cluster randomised trial of a patient decision aid. Themes inductively derived from interviews and observation of consultations with further triangulation with results of decision quality and involvement measurements and case analyses.

**Results:**

The decision aid intervention was employed flexibly within the consultation with both the patient and clinician active in marshalling elements. The decision aid improved processing and organization of information needed for decision making within the consultation interaction. It also improved decision quality by preparing the patient for active involvement within the clinical consultation.

**Conclusion:**

The intervention was acceptable, flexible and readily implemented in primary care consultations. The decision aid was effective in facilitating cognitive processing. The intervention also facilitated rehearsal in preparation for active roles in a shared decision process.

**Trial registration:**

Trials Register Number: ISRCTN14842077. Date registered: 24.06.2010.

## Background

The concept of shared decision-making between clinicians and patients is valued in health care systems around the world [1,2]. An increasing range of interventions that improve shared decision-making have been developed and evaluated in the last decade [3]. The main focus has been patient decision aids (PDAs) designed to help patients reach an informed decision about what is best for them as an individual in the face of treatment choices. These PDAs can function in different media, such as a booklet or web-based programme, which the patient works through, either individually or with support and discussion with their clinician. A systematic review of 86 randomised trials of decision aids concluded that PDAs improve patient knowledge, involvement and comfort in decision making [4]. It is less clear what effects these interventions have on patient-clinician interaction and which components are the essential ingredients for improving decision processes and outcomes [4].

Decision aids are a complex intervention with several interacting components, entailing complex behaviours and a range of effects [5]. In view of their complexity, a degree of flexibility in their implementation is inevitable. The key intervention elements, within even a robust evaluation, may not be readily apparent from trial outcomes data. Further studies to illuminate intervention processes and contexts are therefore useful [5].

Type 2 diabetes is a common long-term condition across the world [6]. In England nearly five per cent of adults have this condition and the prevalence is increasing [7]. It is usually controlled initially with lifestyle changes (diet and physical activity) and oral medications [8]. However, with disease progression, it is necessary to consider other treatment options, such as insulin therapy, to achieve glycaemic control. Patients often find it difficult to decide whether or not to start insulin [9,10]. The decision requires, for example, deliberation about the benefits of improved glucose control with insulin against its side effects such as the risk of hypoglycaemia and weight gain.

The PANDAs (Patients ANd Decision Aids) Trial evaluated a PDA for patients with diabetes facing decisions about treatment options, including insulin, in a primary care setting (NIHR National Trials Register Number 14842077). The components of the PDA are summarised in Outline of PANDAs Decision Aid. The intervention design drew on the Ottawa Decision Support Framework (ODSF), which identifies determinants of health care decisions potentially modifiable by the use of a decision aid [11,12]. The ODSF is based on a range of established cognitive psychological theories and models to provide a pragmatic model for decision aid development [13,14]. The PANDAs PDA contains four contents integrated within a booklet: first, reviewing information about type 2 diabetes, including its symptoms and treatment options; second, evidence-based estimates of the benefits and risks of each treatment option personalised for the individual patient; third, questions to help patients clarify their values, preferences, and expectations as regards these options; and fourth, systematic guidance through the decision-making process.

### Outline of PANDAs Decision Aid

Starting Insulin: Your Choice

Introduction to the decision aid (pages 1–2)

Information (pages 2–6)

• Is there a need to start insulin?

• What happens when people take insulin?

• What are people concerned about when they start insulin?

• How is diabetes affecting you?

• Do you find it difficult to follow diabetes advice*?

• What are your choices?

Step 1: Learn about the choices (pages 7–10)

• Make no change

• Follow diabetes advice more regularly

• Add insulin

• Summary of the three choices

Step 2: Thinking about what is important to you (page 11)

Step 3: What else do you need to make a decision (page 12–13)

Step 4: What are the next steps? (page 14)

Notes

* the lifestyle and oral medications advised

The PANDAs Trial aimed to evaluate the clinical effectiveness of the PDA in improving decision quality (decisional conflict, knowledge, risk perception and involvement in decision making) and the feasibility of implementing the PDA in NHS primary care. A cluster randomised controlled trial was undertaken in England involving 49 general practices and 175 patients [15].

This paper describes a nested mixed methods study undertaken within the intervention arm of the PANDAs Trial of a PDA for people with type 2 diabetes. The nested study aimed to investigate the experiences of using a PDA in routine primary care practice and to explore how components within this 'complex intervention' influenced the decision-making process.

## Methods

The nested study drew on guidance [5] and reviews [16] of methods for embedding a qualitative process study within a clinical trial. A qualitative design has origins in social research and typically entails a more emergent, flexible and inductive approach in contrast to trial methodology. For example, as described below, our qualitative approach included purposive sampling and inductive data analyses.

In addition the design drew on a pragmatic mixed methods approach [17,18] in integrating and combining quantitative measures, from the main trial, where these were germane to the process study aims. A pragmatic approach is an established philosophical and theoretical underpinning for social research, as employed in health services research, where the purpose is primarily policy relevant findings. Data integration is described below. It included, for example, the integration of qualitative data derived from interview and observation transcripts with quantitative data derived from scores of a validated standardised measure of decision conflict.

Following preliminary analyses data were integrated at the level of cases with a view to developing a fuller understanding of the decision aid intervention in the context of its implementation. Each case was a unique instance of a patient and practitioner employing the decision aid in a consultation and integration of data included a variety of types of data and time points of data collection to provide insight on the intervention process. Matrices and threads of case evidence were employed over several iterations. Initial themes were tested and refined within and across case data [19,20]. For example, in testing and refining a theme on how the decision aid was employed to foreground a decision making agenda in the consultation. Analyses were further strengthened by identifying points at which multiple sources of data converged to support findings (triangulation) [20]. For example, where data from observation illustrated an initially passive patient becoming more autonomous within the consultation and this change was also found in the before and after control preference scoring.

The main trial was undertaken in South Yorkshire in England [15]. Participating practices screened computerised diabetes registers for eligible patients with type 2 diabetes. People with type 2 diabetes taking at least two oral glucose lowering drugs at maximum tolerated dose and with a latest HbA1c > 57 mmol/mol in the preceding six months were included in the study. Exclusion criteria included those currently using insulin therapy or people who would have difficulty participating in a study for reasons of language, mental health or cognitive impairment. Patients were initially contacted by letter to participate in the main study and, once they had agreed to take part, were subsequently invited to also join the process study.

Following research ethics approval from North Sheffield Research Ethics Committee, eight pairs of patient-clinician dyads were recruited from the PANDAs Trial intervention arm. Recruitment followed a purposive sampling strategy which aimed to include patients from varied backgrounds, practice contexts and stages of decision-making [21]. In parallel, the patients’ primary care clinician for diabetes care (general practitioner or practice nurse) was also recruited into the study.

Recruited patients to the process study completed a baseline questionnaire (as in the main trial) and then systematically worked through the PDA with the researcher who provided clarification on any issues raised. This was followed by a consultation with a clinician in which the PDA was available to be used by the patient and clinician. Decision quality was determined using a patient self-administered questionnaire following the consultation. Two validated measures of decision quality were employed. 'Decisional Conflict Scale' measures the degree to which the patient feels uncertain about their decision; a low score indicates less decisional conflict [22].’Control Preference Scale’ indicates patients’ preferred role in the decision making process; autonomous means patients are more involved in decision making while passive means less involved [23].

Observation was undertaken via audio recording of the consultations. Verbatim transcripts of the clinical consultation were analysed for themes and also scored using a validated scale for observing patient involvement - the OPTION scale [24,25]. This scale was developed originally in general practice and is designed to measure the extent to which clinicians involve patients in decision making during consultations. To derive a score for the level of involvement, two researchers from the research team independently scored each consultation and then in discussion arrived at an agreed score for each item and total score.

Semi-structured individual interviews with the patient and clinician were conducted separately within two weeks of the consultation (Table 1). The researchers also made field notes on the practice and consultation contexts. The qualitative interviews followed a semi-structured topic guide with open-ended questions to explore the patient’s and clinician’s views of the PDA and the process of its use in practice. All qualitative interviews were audio recorded, transcribed verbatim and QSR NVivo software (version 8) was used to facilitate data management [26].

**Table 1:** Interview guides

**Patients**	
	Could you describe your experience of using the decision aid?
	• Probes to explore perceptions of format, language, understanding and most useful sections
	Was the decision aid useful in helping you to make a decision about your treatment?
	• Probes to explore perceptions of contribution to decision making process
	Could you describe your consultation with the doctor/nurse?
	• Probes to explore use of decision aid within the consultation and contribution to discussion
	Is there anything else that may have helped/hindered with your decision?
	Do you have any suggestions for how we can improve the decision aid in the future?
**Healthcare professionals**	
	Could you describe your thoughts regarding the decision aid?
	• Probes to explore perceptions of most/least useful sections
	Could you describe how the consultation went with the patient?
	• Probes to explore use of decision aid within the consultation and contribution to discussion and patient understanding
	• Probes to compare experiences and difference to the consultation with and without a decision aid
	Is there anything else that may have helped/hindered with the decision?
	Do you have any suggestions for how we can improve the decision aid in the future?

Initial analyses of qualitative data followed the iterative steps established in Framework Analysis [27]. Preliminary coding was refined over a series of iterations to establish themes pertinent to study aims [28]. The qualitative data were then collated with quantitative data, including measures of decision quality, to facilitate case analyses. The final stages of analysis involved triangulation of the data to provide a fuller insight into the experiences of using the PDA and the components that influenced decision making [20,29]. This was an iterative process involving the research team refining and testing the case evidence.

## Results

Eight full sets of qualitative and quantitative data were collected before, during and after a diabetes care consultation in primary care.

### Participants and measures

The patient and clinician characteristics (Table 2) are reasonably diverse (as intended) and reflective of diabetes care programmes in primary care. Three female and five male patients with ages ranging from 39–81 years took part in consultations provided by a mix of clinicians. Control preference measures show a spread of degree of preferred role in decision making across the patients prior to the consultation. Patients E and G preferred a more passive role on these measures; patients C, D and F preferred a more autonomous role.

**Table 2:** Participants with consultation and decision characteristics

**Patient**	**Gender**	**Control preference (pre-consultation)**	**Clinician**	**Consultation (minutes)**	**OPTION score**	**Decision**	**Control preference (post-consultation)**	**Decision conflict (post-consultation)**
A	f	collaborative	PN	20	31	Add insulin	autonomous	0.00
B	f	collaborative	GP	13	15	Current advice*	collaborative	12.50
C	f	autonomous	GP	4	10	Current advice*	autonomous	0.00
D	m	autonomous	PN	17	30	Current advice*	autonomous	25.00
E	m	passive	PN	17	27	Current advice*	autonomous	9.38
F	m	autonomous	PN	29	28	Not sure	autonomous	0.00
G	m	passive	PN	16	34	Make no change	collaborative	25.00
H	m	collaborative	GP	7	15	Add insulin	autonomous	0.00

**Notes**: PN = Practice Nurse, GP = General Practitioner; OPTION (Observing Patient Involvement) score based on 12 item measure, scores range 0 to 48, higher score indicate greater patient involvement in decision taking. *Follow advice about current diabetes therapies (oral medication/lifestyle) more regularly. Decision conflict scores: range = 0–100; low score is better; score below 25 associated with implementing decisions; score above 37.5 associated with decisional delay.

Consultation times ranged between 4 and 29 minutes and these times are typical of diabetes care with longer nurse appointments in comparison to doctors. The OPTION scores indicated a good degree of patient involvement in decision-making but this varied according to the role of the clinician with the nurses involving the patients more than the GPs. Within the OPTION items all clinicians scored best in emphasising the need for a decision and least well on eliciting the patients preferred level of involvement and method for receiving information to help them reach a decision. Across the patients there were a range of decisions reached but generally a low level of decisional conflict following the consultation - indicating a greater likelihood that the decision would be implemented.

These participant, consultation and decision measures also showed good typicality and diversity (as intended) in relation to the main trial intervention arm. For example, mean patient ages and consultation times were almost identical (65.3 years and 15.4 minutes in the cases; 64.5 years and 15.3 minutes in intervention arm); and the treatment decisions reflected the full range of decisions in the intervention group with low decisional conflict scores.

### Consultation processes

Extracts from consultation observation data are presented in Table 3 to give a flavour of the interactions and to illustrate the decision aid intervention processes. The PDA was referred to within all the consultations but varied greatly as to whether it was in the background or foreground (explicitly part) of discussions. There was an on-going process of arriving at a shared sense of whether the PDA should be in the foreground or background. Within this process two influences were evident. First, how imminent an important decision was going to be taken (illustrated in Patient A). If it could be reasonably put off the PDA was left in the background (Patient B). Second, how open minded the patient remained about what to do as they took part in the consultation. If a decision had become clear prior to consultation the PDA was clearly of less value and moved to the background in discussions (Patient H) during the consultation. It was otherwise variously marshalled in support of arguments or to set the agenda or change the subject (Patients E, G and D), particularly within longer discussions of lifestyle factors and diabetes.

**Table 3:** Consultation extracts

**Patient A**	**Nurse** OK. We’ll have a look at [PDA] together then and then we’ll go back onto the insulin things.
	**Patient A** Yeah, yeah. [Discussion covering main sections of the PDA, then:]
	**Nurse** Great, yeah. Clearly understood what we’re talking about.
	***Patient A*** *Yeah, yeah.*
	**Nurse** And these are the choices that you’ve got haven’t you?
	***Patient A*** *Yeah, yeah, they are, yeah.*
	**Nurse** So having looked at those choices and taking on board that, you’ve come to a decision -
	***Patient A*** *Well I think I’ll agree to listen to your concerns as well, you know, because I think you’ve more experience, you know, in sort of dealing with this thing than I have, you know, so if it’s necessary that I need to go on insulin I’m quite prepared to.*
**B**	**GP** How are you getting on?
	**Patient B** I still don’t think I’m any closer to making a decision. I didn’t think I were near enough for that decision yet.
	**GP** So I think we’ve maybe got a bit of scope to increase your medication […] I don’t think you’re absolutely ready to be referred as yet …
**C**	**Nurse:** How did you find that [PDA], useful?
	***Patient:*** *Yeah, a couple of times it’s been mentioned to me about going on insulin.*
	**Nurse:** Mmm, yes, I know.
	***Patient:*** *And I’ve always feared it, you know, I’ve feared it*
**D**	**Nurse** And obviously this [PDA] has given you something to think about.
	***Patient D*** *It doesn’t do a lot for me actually. No. I mean the difference between 22 and 29 -*
	**Nurse** But think about – yeah, but if you were one of those three it would be a big difference.
	***Patient D*** *Yeah, but one of those three could be run over by a bus. I mean that’s what life’s all about. These are probabilities, you know. […] I’m not going to fight statistics.*
**E**	**Nurse** This [PDA] how have you found it? …
	***Patient E*** *Ok. You know, I'm fairly positive with most things.* [discussion follows with reference to diabetes treatments as structured in PDA]
	***Patient E*** *So it's my diet that's got to be sorted a bit better.*
**F**	**Patient F**: But I just do feel that one of the reasons the tablets are struggling is because all the things I’m not doing - the stuff we discussed earlier [in the PDA].
	**Nurse**: That’s it it’s got to be a bit of everything hasn’t it. It’s got to be the correct medication. Diet. Exercise.
	**Patient F**: I’m gonna go back onto the eating regime I was on in 2002 when I lost all the weight.
**G**	***Patient G****: But I'm finding tablets ok you know.*
	**Nurse**: But ok in what way? Because so far they've not brought your sugar level down…
	***Patient G****: They've not made me feel badly at all.*
	**Nurse**: And at the moment what do you think the best plan of action would be? […]
	***Patient G****: Definitely diet yeah.*
	**Nurse**: Adapting that a little better, try and get your sugar levels down.
	**Nurse**: [later…] Just going back to this [PDA], is it important for you to get your sugar down?
**H**	**GP** And really I think we’re going to, you know, need insulin to…
	**Patient H** Yeah, that’s fine.
	**GP** … get it …
	**Patient H** Yeah, let’s get on
	**GP** …nicely under control. Err,… [later…]
	**GP** I’m sure they have…
	**Patient H** Yeah. Been through it.
	**GP** …been through all that. What did you think of the [PDA], was it…?
	**Patient H** Yeah, it was good, yeah.

The information about symptoms and background knowledge of diabetes was referred to more than information quantifying risks. Where the latter became part of the consultation it was not thought to influence decisions (Patient D). Other components were employed to keep a focus on the need for a decision and to broadly revise options.

### Perceptions of intervention process

In all cases, the patients were engaged in using the PDA prior to the consultation and it was referred to by both the patient and clinician in all the consultations. The PDA was perceived to be valuable by both patients and clinicians and they found it particularly useful in terms of the ease of navigation and its full coverage of information (Table 4). The general perception, particularly among the clinicians, was that the decision aid booklet helped influence the decision (Table 4). For example, Patient G and his clinician clearly identified the decision aid as helpful to reach a decision not to start insulin. However, half of the patients saw the PDA as informative but less directly influential on decision-making. For example, Patient E, continued to defer to professional advice.

**Table 4:** Perceptions of the decision aid process

	**Patient**	**Clinician**
A	I think that’s [the PtDA] let me know what options that I had got, yeah.	she picked up very clearly on your charts that were here […] clearly this booklet has given her the indication and helped her make the decision
B	You’ve got it down in writing that and then it’s shown clearly across the board. So I think that’s very effective as well. I really think it, it concentrates your mind […] I think it makes you think in depth about what you really think about it, and you don’t just react	Very [helpful] yes. Yes, it is, and I think you know, like the first couple of pages is really […] I think that could be rolled out to more of the patients
C	I’d made the choice to myself, that I were going on this insulin, so I had to see the doctor […] I confirmed it to myself I was going on it	I think it probably reinforced that he could feel better if his diabetic control was better
D	I’d made my mind up on this, not 100 per cent, but I’d more or less made my mind up months and months ago that this was the way I wanted it to go, otherwise I’d have been on insulin a long while back	I think it’s fantastic, I really, really do. Not only for the patient, but for the professional actually going through a consultation. […] It’s given him a lot of food for thought and I think equipped him with the right knowledge to go away and make that decision
E	So no matter what this book says at the end of the day, professional advice is the thing that you really need	I think it’s highlighted to him the problems […] seeing the pictures and the percentages may have stuck in there a little bit […] in this particular patient, I don’t think it’s added anything to the consultation, knowing him as I do
F	it wouldn’t have persuaded me to not take insulin, but I, as I say, I’ve learnt things	Well, definitely [helpful] because previously erm, when insulin’s been mentioned, it’s no [he wouldn't focus on it as an option]
G	Well it helped me to make a decision […] It told me I didn’t need to go on insulin.	I think the fact that we'd actually got something to show him some sort of card, copy of questions relating to him, you know, tailored to his needs and also then my presentation which perhaps just reinforced everything. […] It's perhaps not the decision we wanted him to make, but that may come in future

Integrating qualitative and quantitative data refined understanding of individual processes in implementing the decision aid (Tables 2, 3, and 4). Patient A made a decision she was likely to implement within a typical length nurse consultation. The PDA was employed explicitly to structure the discussions and the consultation scored highly on patient involvement. In contrast Patient H made the decision to add insulin prior to the consultation. The consultation with a GP was shorter and less involving and the PDA was not used to structure discussions but referred to as a completed step that supported the decision. For both Patient A and B a revision of symptom control and general information about diabetes complications appeared to influence their decision making more than other components in the PDA.

Other patients (B, C, D, E and G) made decisions to follow current advice more carefully or not change treatments. The PDA was variously marshalled by both patient and clinician within the interaction as part of discussions to set a shared agenda or to support a point of argument. For example, Patient G was more passive in their control preference prior a highly involving consultation with a nurse and also scored more highly on the decisional conflict scale. The PDA moved in and out of the consultation discussions marshalled more by the nurse with respect to glucose control and more by the patient with respect to lifestyle approaches to control and symptoms. Agreement was reached (via the PDA) that current symptoms were related to diabetes control and that steps to adapt diet should be taken.

Patient E remained unsure as regards the decision options after a long and highly involving consultation with a nurse. This individual with an autonomous control preference also scored low on the Decisional Conflict Scale. The consultation data indicated that the PDA was fully employed and in the same manner as for other patients (Table 3). Indeed a decision to lose weight was apparently reached after discussing treatment options during the consultation.

### Active components

Three themes with respect to active components are presented in Table 5 with supporting data. First, the PDA raised awareness of the key issues in the choice of treatment, second, it improved the knowledge necessary for making the decision; third it provided preparation for involvement in the consultation. The health information relating to poor diabetes control and current symptoms was valued and had an impact on the decision-making process. Several patients seemed unaware of their symptoms related to diabetes. The provision of personalised information about morbidity risks of poor diabetes control was valued by the patients. However, information about the risks and benefits of each treatment option, including the numerical risks and pictorial presentation, had less impact on the consultation interaction (see below). Patients valued their involvement in decision making and the personalised care (‘what the decision could mean for me’) which they received from the clinicians. The PDA was also thought to have prepared the patient for the consultation, providing a shared terminology and agenda. In this respect the PDA was seen as a tool to help the patient and clinician prioritise the agenda and focus on an issue that might otherwise be lost within a general discussion.

**Table 5:** Perceptions of active components

**Theme**	**Illustrative quotes**
Challenging preconceived ideas	I think it really highlights the problems that they are experiencing is due to their poorly controlled diabetes, whereas previously I think they just would have thought, oh it’s because I’m getting older [Interview E Clinician] what do you think of straightway, he’s a druggie, that’s the last thing I want to be even thought of, so there’s that side of it [Interview D Patient on preconceptions of insulin therapy]
Increasing knowledge base	I think she had a better understanding. She certainly was more aware of complications with diabetes. [Interview A Clinician] but I didn’t realise that you could get heart disease through diabetes, you know [Interview A Patient] I think if you don’t use the decision aid it’s very sort of doctor looking at figures saying ‘I’m going to have to refer you’ and then it’s like they don’t know why and all they know is that we’re referring you. So I think when you use the decision aid I think they actually have much more understanding [Interview B Patient]
Rehearsal and prompt for agenda setting and structuring of the consultation	It has guided my consultation into a more, probably more thorough and more focused path to what it was before [Interview D Clinician] It helped me to understand where she's coming from. [Interview E Patient] I mean he had his open on the desk. So that was sort of an indirect way of letting me know that he was quite happy to look at it [Interview G Clinician]

## Discussion

Our mixed methods study nested within the PANDAs Trial has usefully triangulated qualitative and quantitative data to explore active elements within a complex decision aid intervention. Strengths of the study are that PANDAs is a typical decision aid intervention and that, within a pragmatic trial, an intervention implemented in typical health care settings. However, the small sample sizes in the nested process study mean our findings should be viewed as provisional. They are nevertheless interesting and add to understanding of a complex intervention. The findings support aspects of the cognitive underpinnings for decision aid interventions but also point to other active ingredients that will require further research to fully be illuminated.

The main trial had shown that the PANDAs PDA is an effective intervention for general practice and its use leads to improved decision-making outcomes for patients [15]. The nested study adds evidence of a practical and flexible intervention readily incorporated into routine practice to facilitate shared decision-making within a consultation. There are insights as to how both patient and clinician can be active in employing its components to facilitate a shared decision [30].

The PANDAs PDA clearly improves the understanding and organization of information during the consultation by allowing the patient and clinician to consider and summarise a range of objective and subjective information. The elements for them to consider within the decision were, therefore, easier to remember and focus on in the consultation. However, information about the numerical risks and benefits was not apparently integrated into the decision-making. Rather, more descriptive information of the possible links between current symptoms and glucose control was more influential. In this respect the PDA may improve but not substantially alter the broad heuristic thinking that typically underpins decision making by patients [3].

Most patients with type 2 diabetes have various other issues aside from glucose control that require attention within the consultation. The PDA helped by preparing patients for the likely structure and agenda of the consultation – thereby enabling better involvement in decision making. It also appeared to support a more involving style of consultation encouraging the patient to be more active in setting the agenda in the interaction. In this respect the PDA is an intervention facilitative of communication styles that encourage patient agenda setting and involvement in decisions [31,32].

Whilst information processing and consultation structuring are clearly active ingredients, other non-cognitive elements may also be active. For instance, detailed numerical information appears to be liked by patients as an indicator of personalised care and less for its value in actually estimating risks. In effect it works more as social learning than cognitive processing. Social learning draws attention to observation and modelling as motivators of a behaviour or action [33]. In contrast cognitive theories emphasise internal information processing and the logical sifting of thoughts to arrive at a decision. Personalised risk information is active in the intervention because it rewards motivation to take part in a social decision making process; it is less apparently active because it facilitates information processing.

Broadly, therefore, our study supports the underlying cognitive theoretical models for decision aids [13,14] but suggests also their value as social encouragement to share in a decision process and as preparation for active involvement within the consultation interaction. The simple presence of a tool provides an expectation of involvement and a more shared agenda which, in turn, alters behaviour. The OPTION scale has been used with clinicians in primary care previously [24,25]. Only cautious comparisons are appropriate but it is interesting to note that the scores in these other studies were substantially lower than that observed in this study of a decision aid in use. This adds to the evidence that a decision aid facilitates involvement in the consultation by both parties and contributes to greater autonomy and satisfaction with the decision by the patient [4].

Wider implementation of PDAs remains a challenge [34]. Shared decision making is not the norm as yet in most health care systems despite efforts to improve involvement and patient self-management [35-37]. A number of qualitative studies have explored user and provider perceptions of implementing decision aids in primary care practice [38-41]. As in our study there is broad support from patients for the educational and informational value of decision aids but greater reservations from healthcare providers with respect to decision aid costs and practicalities. Ease of navigation and other design features are critical to acceptance; as are other factors affecting consultation time and ease of integration into routine practice [38-41]. Involvement of clinicians and patients in the development stages of a PDA is crucial and this element is emphasised in International Patient Decision Aid Standards [42].

Another issue is how to present risk information in an acceptable and engaging form for patients and how this may influence decision making [43]. The PANDAs PDA used numbers, words and pictures [44] to convey information about the absolute risks of diabetes complications over five years. The nested study suggests this information had a mixed reception and did not directly influence decision making in the way that was anticipated.

Further research should examine which cognitive and social components of a PDA are most useful with which category of patients [30]. Patients found the personalised nature of the PANDAs PDA helpful and it reinforced a sense of personalised care and therefore commitment to shared decision making. Further study of how this and other design elements can be integrated into current clinical practice would be useful [45], particularly for understanding how an intervention may be more successfully implemented in different contexts. For example, would an electronic version of the PANDAs PDA (that can be maintained with the electronic patient record) be as effective?

Policy makers, professional leaders and patient groups would all like to see more effective involvement of patients in their own care [46,47]. The PANDAs PDA can be recommended for general practice, particularly within the context where a decision is imminent. For a patient who is already confident and autonomous as regards health care decision making, the PDA can be used within a standard length consultation. For patients who are less engaged as regards to decision-making, or feel much undecided, the PDA should be used in conjunction with a longer and more involving consultation. The PDA can be used to organise complex information and prepare patients for agenda setting within the consultation. It can be used to reinforce a sense of personalised care, to structure the decision making communication as well as to focus on and revise current symptoms. All of which are active ingredients in helping patients to reach a decision.

## Conclusions

Decision aids that can be employed actively and flexibly by both patients and clinicians within the consultation interaction are helpful for implementation. Their design should of course attend to the information processing needs of patient and clinician. Further research about other active intervention elements of motivation and social learning for a shared social process would be useful.

## Competing interests

The authors declare that they have no competing interests.

## Authors’ contributions

All authors were involved in substantial contributions to study conception and design and to data collection, analysis and interpretation. IB and AB carried out drafting the article and all authors were involved in revising it critically for important intellectual content and approving the final version to be published. All authors read and approved the final manuscript.
